# Development and validation of a prediction model for adenoma detection during screening and surveillance colonoscopy with comparison to actual adenoma detection rates

**DOI:** 10.1371/journal.pone.0185560

**Published:** 2017-09-28

**Authors:** Eelco C. Brand, Julia E. Crook, Colleen S. Thomas, Peter D. Siersema, Douglas K. Rex, Michael B. Wallace

**Affiliations:** 1 Department of Gastroenterology and Hepatology, Mayo Clinic, Jacksonville, Florida, United States of America; 2 Department of Gastroenterology and Hepatology, University Medical Center Utrecht, Utrecht, the Netherlands; 3 Division of Biomedical Statistics and Informatics, Mayo Clinic, Jacksonville, Florida, United States of America; 4 Department of Gastroenterology and Hepatology, Radboud University Medical Center, Nijmegen, The Netherlands; 5 Department of Gastroenterology and Hepatology, Indiana University Medical Center, Indianapolis, Indiana, United States of America; University Hospital Llandough, UNITED KINGDOM

## Abstract

**Objective:**

The adenoma detection rate (ADR) varies widely between physicians, possibly due to patient population differences, hampering direct ADR comparison. We developed and validated a prediction model for adenoma detection in an effort to determine if physicians’ ADRs should be adjusted for patient-related factors.

**Materials and methods:**

Screening and surveillance colonoscopy data from the cross-sectional multicenter cluster-randomized Endoscopic Quality Improvement Program-3 (EQUIP-3) study (NCT02325635) was used. The dataset was split into two cohorts based on center. A prediction model for detection of ≥1 adenoma was developed using multivariable logistic regression and subsequently internally (bootstrap resampling) and geographically validated. We compared predicted to observed ADRs.

**Results:**

The derivation (5 centers, 35 physicians, overall-ADR: 36%) and validation (4 centers, 31 physicians, overall-ADR: 40%) cohort included respectively 9934 and 10034 patients (both cohorts: 48% male, median age 60 years). Independent predictors for detection of ≥1 adenoma were: age (optimism-corrected odds ratio (OR): 1.02; 95%-confidence interval (CI): 1.02–1.03), male sex (OR: 1.73; 95%-CI: 1.60–1.88), body mass index (OR: 1.02; 95%-CI: 1.01–1.03), American Society of Anesthesiology physical status class (OR class II vs. I: 1.29; 95%-CI: 1.17–1.43, OR class ≥III vs. I: 1.57; 95%-CI: 1.32–1.86), surveillance versus screening (OR: 1.39; 95%-CI: 1.27–1.53), and Hispanic or Latino ethnicity (OR: 1.13; 95%-CI: 1.00–1.27). The model’s discriminative ability was modest (C-statistic in the derivation: 0.63 and validation cohort: 0.60). The observed ADR was considerably lower than predicted for 12/66 (18.2%) physicians and 2/9 (22.2%) centers, and considerably higher than predicted for 18/66 (27.3%) physicians and 4/9 (44.4%) centers.

**Conclusion:**

The substantial variation in ADRs could only partially be explained by patient-related factors. These data suggest that ADR variation could likely also be due to other factors, e.g. physician or technical issues.

## Introduction

Colonoscopy combined with polypectomy, when necessary, has been shown to decrease colorectal cancer (CRC) incidence[1] and CRC-related mortality.[1,2] However, the protective benefit is reduced by the occurrence of interval-CRC, i.e. CRC occurring within the colonoscopy surveillance interval. Three main reasons for the occurrence of interval-CRC have been suggested in literature, namely: 1) missed lesions during colonoscopy (accounting for approximately 50–60% of the cases), 2) incomplete resection, and 3) newly developed cancers.[3] The proportion of patients undergoing colonoscopy in which at least one adenoma is detected, the adenoma detection rate (ADR), has been shown to be inversely associated with the development of interval-CRC.[4,5] Quality improvement in colonoscopy therefore aims, among other things, at increasing and thereby achieving sufficient physicians’ ADRs. American and European guidelines recommend thus ADRs to be ≥25%,[6,7] i.e. ≥30% in male and ≥20% in female patients.[7]

The influence of several modifiable factors, such as procedural and technological factors, on ADR have been studied.[8] Moreover, training programs to improve the ADR have been developed and evaluated.[9,10] Nevertheless, ADRs vary widely between physicians,[11] possibly caused by patient population differences. ADR comparison between centers and physicians is therefore challenging and even more complicated by the strict domain in which the ADR should be calculated, i.e. for patients at average risk of adenoma detection in a screening population. Average risk individuals are those without additional risk factors for adenoma detection, e.g. a family history of CRC, a personal history of CRC or colorectal adenomas, and are not preselected for colonoscopy through, for example, stool tests, e.g. fecal immunochemical tests, or other diagnostic tests. Several initiatives aim to provide physicians with feedback on their ADR through online databases, such as the Gastro-Intestinal Quality Improvement Consortium (GIQuIC).[12] The feedback could be improved if next to these unadjusted ADRs the expected ADR for a physician’s patient population could be predicted.

Several prediction models based on patient risk factors have been developed for the detection of advanced adenomas[13–23] and a few for any adenoma.[18,20,23–25] Most of the models for any adenoma detection were developed in Asian populations,[18,20,23,24] with a generally lower adenoma prevalence than Western populations, had a moderate discriminative ability, and only included screening colonoscopies.[18,20,23–25]Application of these models is thus hampered in a Western setting and in day-to-day practice where screening and surveillance colonoscopies might both be used for ADR calculations as they are performed side-to-side.

Therefore, the aim of the present study was to develop and validate a prediction model for colorectal adenoma detection based on patient risk factors in a screening and surveillance population. The secondary aim was to compare the observed individual physicians’ and centers’ ADRs to the predicted proportion of patients with ≥1 adenoma based on the developed model.

## Materials and methods

The present study has been performed and reported according to the TRIPOD statement for the reporting of multivariable prediction models (S2 File).[26]

### Data source

We used the data from the cluster-randomized cross-sectional Endoscopic Quality Improvement Program-3 (EQUIP-3) study (Clinical Trials Registration: NCT02325635) that ran from September 2013 until January 2015.[9] The EQUIP-3 study was approved by the Mayo Clinic Institutional Review Board and was considered minimal risk and exempt from patient-level consent.[9] In the EQUIP-3 study centers were randomized, after a lead-in phase, to receive a quality improvement program aimed at increasing the ADR or no intervention. During the lead-in phase data on all colonoscopies performed in all participating centers was collected enabling analyses of the colonoscopy quality metrics without the influence of the quality improvement program. The centers randomized to receive the quality improvement program, received the first EQUIP training as described in the EQUIP-1 study,[27] consisting of baseline measurement of ADR, followed by an in-person 1-hour powerpoint based training emphasizing on improvement of adenoma detection and flat lesion recognition. Furthermore in these centers posters about EQUIP were placed in each endoscopy room, and the ADR of all endoscopists was one-on-one discussed, typically with low performers. Each center and individual then received regular follow-up ADR reports, approximately monthly during the post-intervention phase.[9] Data was collected through the GIQuIC-form (S1 File)[12] by the physician or nurse at the end of the procedure. Pathology results were entered subsequently when available. Predictor assessment was thus blinded for the outcome.

### Sample split

The original dataset was split into two cohorts based on the performing center. This enabled validation of the model in geographically different centers, which is a second-best after fully external validation. In short, in the first cohort, i.e. the derivation cohort, the model will be developed and internally validated. Subsequently, the fitted model will be geographically validated in the second cohort, i.e. the validation cohort. The number of patients, centers, physicians, and randomization to the intervention were balanced between the derivation and validation cohort.

### Participants

Sixty-six physicians from nine centers in the United States, i.e. within California, Illinois, Indiana, New Mexico, New York, Ohio, Tennessee, and Virginia, participated. Patients undergoing outpatient colonoscopy were included in the EQUIP-3 study if 1) they did not have a history of colorectal surgery; 2) indication for colonoscopy was screening or surveillance, as indicated in the GIQuIC database; and 3) bowel preparation was adequate, i.e. sufficient to accurately detect polyps ≥6 mm.

In the present study we excluded patients: 1) <50 years, because screening is recommended ≥50 years; 2) with a known increased risk of colorectal neoplasia who are normally discarded in ADR calculations, i.e. colonoscopy for a high-risk genetic CRC syndrome or surveillance colonoscopy because of inflammatory bowel disease; and 3) a personal history of CRC, because these patients will probably have undergone colorectal surgery. Screening and surveillance colonoscopies were both included, because these are performed side-to-side in daily practice and might both be used in ADR calculations. Furthermore, a prediction model for adenoma detection in both a screening and surveillance population potentially facilitates ADR comparisons across physicians and centers in both screening and surveillance settings.

A minuscule proportion (N = 53, 0.2%) of colonoscopies were probably performed in a patient already included in the study, we therefore excluded these second colonoscopies from the database.

### Outcome

The outcome of the prediction model was the detection of ≥1 histologically confirmed colorectal adenoma per patient. The histological assessment was performed in daily practice, and thus not completely blinded for patient factors, e.g. sex and age of the patient. However, the pathologist did not have access to the GIQuIC form and was consequently blinded to factors such as BMI, ASA class, and race and ethnicity, and it is therefore unlikely that these factors could have influenced the pathologists’ judgment.

### Predictors for adenoma detection

Pre-colonoscopic possible predictors for adenoma detection were selected based on previously published prediction models for the detection of (advanced) adenomas.[13–25]

Age and body mass index (BMI) in kg/m^2^ were analyzed as continuous variables. We categorized race as: “African-American”, “Asian”, “other” (due to the small numbers of patients per subgroup including white, native American, Alaska native, native Hawaiian, native Pacific and patients categorized as other), and “unknown or patient declined to provide”. Ethnicity was categorized as: “Hispanic or Latino”, “not Hispanic or Latino”, and “unknown or patient declined to provide”.

As a proxy for clinical condition and co-morbidities the American Society of Anesthesiology physical status (ASA) was used[28]. Because no ASA V patients and only a small number of ASA IV patients were included, we categorized this variable as: “ASA I”, “ASA II” and “ASA III or IV”.

The indication of colonoscopy was surveillance, i.e. a personal history of colorectal adenomas or surveillance marked as indication on the GIQuIC form, or colorectal cancer screening. Family history, i.e. ≥1 first-degree relative <60 years diagnosed with the condition, of colorectal adenomas and family history of CRC were analyzed as dichotomous variables. The pre-colonoscopic risk on adenoma detection, i.e. high or low, was not included as a possible predictor due to possible multicollinearity with indication, family history of CRC and family history of colorectal adenomas.

### Statistical analysis

All statistical analyses were performed with R language environment for statistical computing version 3.1.3.[29]

#### Sample size

We refer to the original paper for sample size calculations.[9] The number of patients with ≥1 adenoma detected exceeded ten per possible predictor considered, and should therefore be sufficient for the analysis.

#### Missing data

To correct for possible errors with data-entry on the GIQuIC-form we recoded the following implausible values as missing: BMI <15 or >55kg/m^2^, height <140 or >220cm, weight <40 or >250kg. We assumed missing data of these variables to be missing at random, in other words the fact that the data is missing is not related to the value that is missing.[30] Multiple imputation based on iterative (10 iterations) chained equations with predictive mean matching was performed, creating 20 multiple imputed datasets, using the MICE-package for R.[31] The multiple imputation procedure was performed based on center, physician, all possible predictors and detection of: ≥1 adenoma, ≥3 adenomas, any polyp, advanced adenoma(s), adenocarcinoma(s), and serrated lesion(s).[32] The imputed values for BMI were calculated from the imputed height and weight.

We assumed patient’s race and ethnicity being categorized as “unknown or patient declined to provide” to be missing not at random, i.e. we expect that there is a reason why these values are not filled out in the GIQuIC database, and we therefore retained this category in the modeling process.

#### Descriptive statistics

The number of patients per center and per physician is presented as medians and ranges. Continuous baseline characteristics are presented as mean ± standard deviation, and categorical data as frequencies with proportions. The proportion of patients with ≥1 adenoma detected, i.e. the ADR, is reported per subgroup.

Univariable odds ratios (ORs) including 95%-confidence intervals (CIs) for the detection of ≥1 adenoma were estimated for all possible predictors based on logistic regression modeling. An odds ratio was regarded statistically significant if the 95%-CI did not include ‘one’.

#### Model development and internal validation within the derivation cohort

A multivariable logistic regression model with detection of ≥1 adenoma as outcome was fitted with the following possible predictors: age, sex, BMI, race, ethnicity, ASA class, indication, family history of CRC, family history of colorectal adenomas, interaction between race and sex, and interaction between ethnicity and sex. The model estimates were adjusted for randomization to a quality improvement intervention by adding the dichotomous variable “colonoscopy performed after intervention received versus no intervention received” to the model. The final model was selected using stepwise backwards selection based on Akaike’s information criterion.

The model’s discriminative ability was assessed with the apparent C-statistic, which is equivalent to the area under the receiver operating characteristic curve. Subsequently internal validation was performed using 1000 bootstrap resamples per imputed dataset to calculate the shrinkage factor and optimism-corrected C-statistic. Regular bootstrap resampling without taking clustering into account was performed.[33] The optimism-corrected model coefficients and intercept were calculated based on the shrinkage factor. The model calibration was visually assessed with a calibration plot.

#### Geographical validation and model-update within the validation cohort

Because we expected differences in the baseline adenoma prevalence and overall effect of predictors due to geographic variation we performed logistic re-calibration by fitting a logistic regression model with the original model’s linear predictor as the independent variable and detection of ≥1 adenoma as the dependent variable. The intercept and coefficient of this new logistic model are the re-calibrated intercept and slope-correction, respectively.[34] The discriminative ability (C-statistic) and calibration (with a calibration plot) of the updated model were assessed.

### Predicted proportion of patient with ≥1 adenoma detected compared to actual ADRs

In both cohorts the probability for the detection of ≥1 adenoma per patient was predicted with the final model. The sum of the predicted probabilities of adenoma detection per patient was calculated resulting in the predicted proportion of patients with ≥1 adenoma per physician and center. These predicted proportions were graphically compared to the physicans’ and centers’ observed ADR, i.e. the proportion of patients with ≥1 adenoma detected, including 95% Wilson CIs. The observed ADR was considered considerably lower than predicted if the upper bound of the 95% CI was lower than the predicted ADR, and considerably higher than predicted if the lower bound of the 95% CI was higher than the predicted ADR.

## Results

### Study population

**Fig 1:**
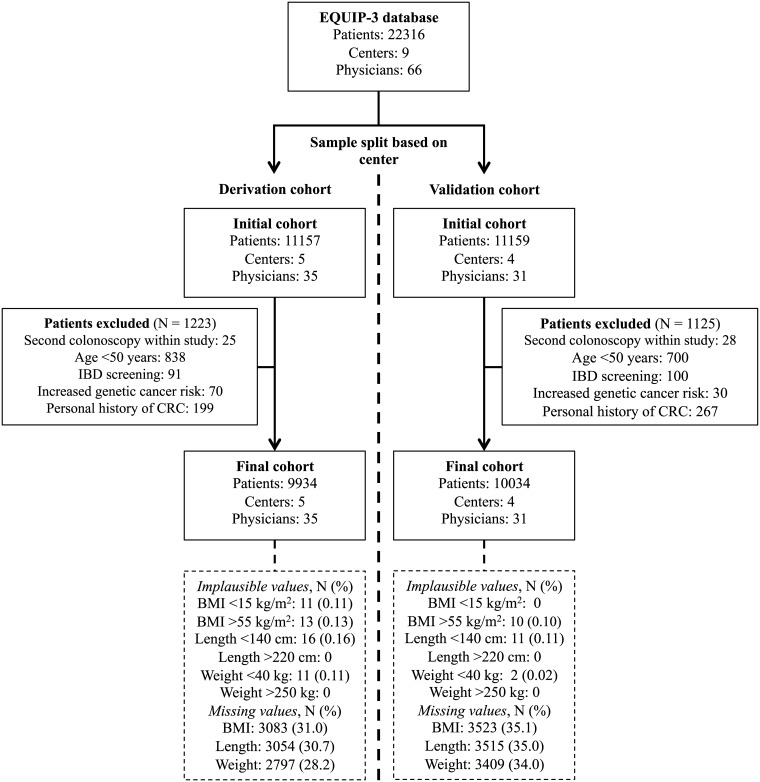
Flowchart of patients in the derivation and validation cohort with an overview of the reasons for and number of excluded patients and number of implausible and missing values. BMI, body mass index; CRC, colorectal cancer; EQUIP-3, Endoscopic Quality Improvement Program-3 study; IBD, inflammatory bowel disease; N, number of patients.

The 22316 patients were divided between the derivation and validation cohort. In the derivation cohort 9934 patients (1223 patients were excluded) were examined by 35 physicians in five centers (Fig 1). In the validation cohort 10034 patients (1125 patients were excluded) were examined by 31 physicians in four centers.

### Missing values

No outcome data were missing. A substantial proportion (28.2–35.1%) of patients had missing values for height, weight or BMI (Fig 1).

### Baseline characteristics and ADR per subgroup of patients

The provider and patient characteristics, and ADR per variable are summarized in Table 1. The reasons for being at high risk of adenoma detection are summarized in S1 Table. Within the derivation cohort the mean age was 60.2 years, 47.6% were male, and the mean BMI was 28.3 kg/m^2^. The overall ADR was 35.9%, with ADRs for female (29.6%) and male (42.9%) patients above the recommended thresholds[7] of ≥20% and ≥30% respectively. Within the validation cohort the patients were slightly older (mean age 61.0 years), almost the same proportion was male (47.8%), and patients had a higher mean BMI (29.2 kg/m^2^). The overall ADR (40.0%) was higher, and again the ADR for female (34.9%) and male (45.6%) patients was above the recommended thresholds[7].

**Table 1:** Provider and patient characteristics for the derivation and validation cohort and the adenoma detection rate per patient subgroup.

	Derivation cohort		Validation cohort	
	All patients [N = 9934] N (%)^a^	Patients with ≥1 adenoma [N = 3568] N (ADR)^a^	All patients [N = 10034] N (%)^a^	Patients with ≥1 adenoma [N = 4013] N (ADR)^a^
**Provider characteristics**				
Center^b^, median N (range)	1544 (1144–3336)	537 (431–1232)	2492.5 (1228–3821)	735 (561–1982)
Physician^c^, median N (range)	289 (55–725)	93 (11–306)	246 (57–812)	96 (12–330)
**Patient characteristics**				
Age in years, mean ± SD	60.2 ± 7.7	61.3 ± 7.8	61.0 ± 8.2	61.8 ± 8.3
Female	5209 (52.4)	1542 (29.6)	5237 (52.2)	1826 (34.9)
Male	4725 (47.6)	2026 (42.9)	4797 (47.8)	2187 (45.6)
BMI in kg/m^2^, mean ± SD	28.3 ± 5.6 [N = 6851]^d^	28.8 ± 5.5 [N = 2538]^d^	29.2 ± 5.8 [N = 6511]^d^	29.7 ± 5.8 [N = 3120]^d^
ASA I	2689 (27.1)	793 (29.5)	1920 (19.1)	668 (34.8)
ASA II	6455 (65.0)	2420 (37.5)	7532 (75.1)	3119 (41.4)
ASA III or IV^e^	790 (8.0)	355 (44.9)	582 (5.8)	226 (38.8)
Race				
Other^f^	6368 (64.1)	2250 (35.3)	8306 (82.8)	3463 (41.7)
African-American or black	1320 (13.3)	477 (36.1)	701 (7.0)	263 (37.5)
Asian	134 (1.3)	49 (36.6)	149 (1.5)	51 (34.2)
Unknown or patient declined to provide	2112 (21.3)	792 (37.5)	878 (8.8)	236 (26.9)
Ethnicity				
Not Hispanic or Latino	5933 (59.7)	2067 (34.8)	8451 (84.2)	3517 (41.6)
Hispanic or Latino	1415 (14.2)	533 (37.7)	87 (0.9)	31 (35.6)
Unknown or patient declined to provide	2586 (26.0)	968 (37.4)	1496 (14.9)	465 (31.1)
Indication for colonoscopy^g^				
Screening	7353 (74.0)	2409 (32.8)	6518 (65.0)	2361 (36.2)
Surveillance	2581 (26.0)	1159 (44.9)	3516 (35.0)	1652(47.0)
Risk assessment^h^				
Average risk	6558 (66.0)	2168 (33.1)	6589 (65.7)	2452 (37.2)
High risk	3376 (34.0)	1400 (41.5)	3445 (34.3)	1561 (45.3)

ADR, adenoma detection rate, i.e. proportion of patients with ≥1 adenoma detected per subgroup; ASA, American Society of Anesthesiology physical status class; BMI, body mass index; N, number of patients; SD, standard deviation.

^a^Unless, stated otherwise in the beginning of the row.

^b^Five centers were included in the derivation cohort, and four centers in the validation cohort.

^c^Thirty-five physicians were included in the derivation cohort, and thirty-one physicians were included in the validation cohort.

^d^Number of patients without missing values.

^e^Only three patients in the development cohort and no patients in the validation cohort were in ASA category IV and therefore ASA III and IV were combined.

^f^Including white, native American, Alaska native, native Hawaiian, native Pacific patients and patient’s race categorized as other.

^g^The indication is considered surveillance for patients with a personal history of colorectal adenomas or surveillance marked as indication on the GastroIntestinal Quality Improvement Consortium (GIQuIC) form.

^h^The number of patients categorized per reason for high risk of adenoma detection are displayed in supporting information Table 1 (S1 Table).

Regarding the baseline characteristics the derivation and validation cohort differed the most with respect to the included proportion of African-American and Hispanic or Latino patients, and colonoscopies with screening as indication (Table 1).

### Univariable association between predictors and detection of ≥1 adenoma

In the derivation cohort statistically significant positive predictors for detection of ≥1 adenoma were age (per year increase), male sex, BMI (per kg/m^2^ increase), ASA class II compared to class I, ASA class III or IV compared to I, Hispanic or Latino ethnicity and surveillance compared to screening as indication (Table 2).

**Table 2:** Univariable odds ratios of possible risk factors for the detection of ≥1 adenoma within the derivation and validation cohort.

Possible predictors	Derivation cohort	Validation cohort
	Univariable odds ratio^a^ [95%-CI]	Univariable odds ratio^a^ [95%-CI]
Age (per year increase)	1.03 [1.02–1.04]	1.02 [1.02–1.03]
Female	1.00 (ref)	1.00 (ref)
Male	1.79 [1.64–1.94]	1.57 [1.44–1.70]
BMI (per kg/m^2^ increase)^b^	1.02 [1.01–1.03]	1.03 [1.02–1.04]
ASA I	1.00 (ref)	1.00 (ref)
ASA II	1.43 [1.30–1.58]	1.32 [1.19–1.47]
ASA III or IV^c^	1.95 [1.66–2.30]	1.19 [0.98–1.44]
Race		
Other^d^	1.00 (ref)	1.00 (ref)
African-American or black	1.04 [0.91–1.17]	0.84 [0.72–0.98]
Asian	1.06 [0.73–1.50]	0.73 [0.51–1.02]
Unknown or patient declined to provide	1.10 [0.99–1.22]	0.51 [0.44–0.60]
Ethnicity		
Not Hispanic or Latino	1.00 (ref)	1.00 (ref)
Hispanic or Latino	1.13 [1.00–1.27]	0.78 [0.49–1.20]
Unknown or patient declined to provide	1.12 [1.02–1.23]	0.63 [0.56–0.71]
Indication for colonoscopy^e^		
Screening	1.00 (ref)	1.00 (ref)
Surveillance	1.67 [1.53–1.83]	1.56 [1.44–1.70]
History of colorectal cancer		
No	1.00 (ref)	1.00 (ref)
Family^f^	0.88 [0.75–1.02]	1.13 [0.97–1.31]
History of colorectal adenomas		
No	1.00 (ref)	1.00 (ref)
Family^f^	1.12 [0.93–1.35]	1.17 [0.88–1.55]

ASA, American Society of Anesthesiology physical status class; BMI, body mass index; CI, confidence interval; ref, reference category.

^a^Odds ratios based on a univariable logistic regression model with detection of ≥1 adenoma as outcome. 95% confidence intervals are profiled confidence intervals.

^b^This association is calculated after multiple imputation.

^c^Only three patients in the development cohort and no patients in the validation cohort were in ASA category IV and therefore ASA III and IV were combined.

^d^Including white, native American, Alaska native, native Hawaiian and native Pacific patients and patient’s race categorized as other.

^e^The indication is considered surveillance for patients with a personal history of colorectal adenomas or surveillance marked as indication on the GastroIntestinal Quality Improvement Consortium (GIQuIC) form.

^f^Family history is defined as a first degree relative diagnosed with the condition at an age <60 years.

Some univariable associations within the validation cohort clearly differed from those observed in the derivation cohort. The OR of ASA class III or IV compared to I was lower and statistically non-significant. The OR of family history of CRC was above 1 in the validation cohort, but was still non-significant. The OR for African-American, Asian, and Hispanic or Latino patients was <1 within the validation cohort, while these variables had an OR >1 in the derivation cohort.

### Model development and validation within the derivation cohort

**Fig 2:**
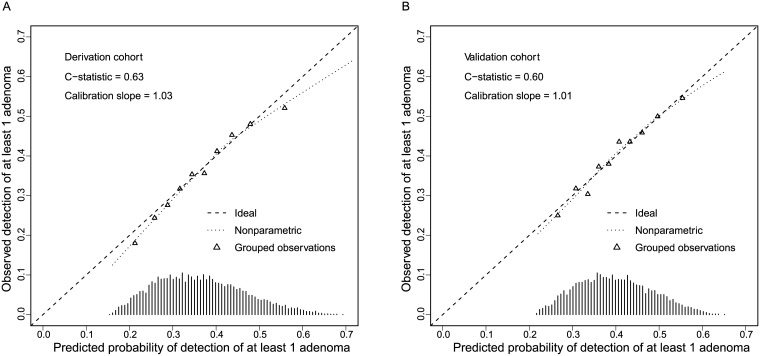
Calibration plot of the model performance A) within the derivation cohort after internal validation (figure at the left), and B) within the validation cohort after logistic re-calibration (figure at the right). Grouped observations are grouped per decile of patients. The observed proportion of patients with ≥1 adenoma detected is displayed on the y-axis, and the predicted proportion of patients with ≥1 adenoma on the x-axis. C-statistic, concordance statistic.

After stepwise backwards selection the following patient-related predictors were selected in the multivariable model (Table 3): age, sex, BMI, ASA class, indication (surveillance versus screening), and Hispanic or Latino ethnicity. The discriminative ability was modest (apparent C-statistic: 0.630 and optimism-adjusted C-statistic: 0.626). Calibration of the model was visually accurate, however, the model overestimated the probability of the detection of ≥1 adenoma among patients with a low observed and especially high observed adenoma detection (Fig 2A).

**Table 3:** Prediction model^a^ for the detection of ≥1 adenoma per patient based on multivariable logistic regression within the derivation cohort.

Factors	Uncorrected multivariable OR [95%-CI]	Corrected^b^ β coefficients	Corrected^b^ multivariable OR [95%-CI]
Intercept	-	-3.134	-
Age (per year increase)	1.02 [1.02–1.03]	0.023	1.02 [1.02–1.03]
Female	1.00 (ref)	0 (ref)	1.00 (ref)
Male	1.76 [1.62–1.92]	0.549	1.73 [1.60–1.88]
BMI (per kg/m^2^ increase)	1.02 [1.01–1.03]	0.017	1.02 [1.01–1.03]
ASA I	1.00 (ref)	0 (ref)	1.00 (ref)
ASA II	1.30 [1.18–1.44]	0.256	1.29 [1.17–1.43]
ASA III or IV	1.59 [1.34–1.90]	0.451	1.57 [1.32–1.86]
Ethnicity			
Not Hispanic or Latino	1.00 (ref)	0 (ref)	1.00 (ref)
Hispanic or Latino	1.13 [1.00–1.28]	0.122	1.13 [1.00–1.27]
Unknown or patient declined to provide	1.16 [1.05–1.29]	0.148	1.16 [1.05–1.28]
Indication for colonoscopy^c^			
Screening	1.00 (ref)	0 (ref)	1.00 (ref)
Surveillance	1.41 [1.28–1.55]	0.332	1.39 [1.27–1.53]

ASA, American Society of Anesthesiology physical status class; BMI, body mass index; CI, confidence interval; OR, odds ratio; ref, reference category.

^a^The presented odds ratios are adjusted for EQUIP intervention (colonoscopy performed after versus no intervention received) which had an uncorrected OR of 1.24 [95%-CI: 1.13–1.36] in the final model.

^b^Corrected after internal validation using bootstrap resampling with a shrinkage factor of 0.969. The intercept is additionally corrected by subtraction of the intercept correction of -0.017.

^c^The indication is considered surveillance for patients with a personal history of colorectal adenomas or surveillance marked as indication on the GastroIntestinal Quality Improvement Consortium (GIQuIC) form.

### Geographical validation within the validation cohort

The re-calibrated model with an intercept of -2.406 and overall slope correction of 0.757 (final model estimates not shown) had a modest discriminative ability (C-statistic 0.603). The model tended to overestimate the adenoma detection among patients with a high observed adenoma detection (Fig 2B).

### Predicted proportion of patients with ≥1 adenoma compared to observed ADR

**Fig 3:**
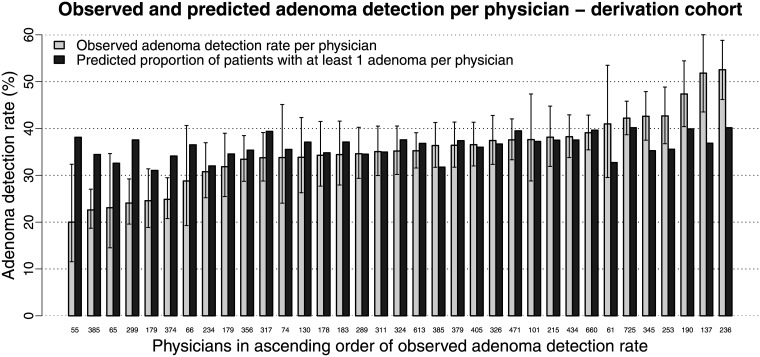
Observed adenoma detection rates (including 95% Wilson confidence intervals) versus predicted proportions of patients with ≥1 adenoma per physician within the derivation cohort. The physicians are ranked in ascending order of adenoma detection rate. The numbers on the x-axis denote the number of patients per physician.

**Fig 4:**
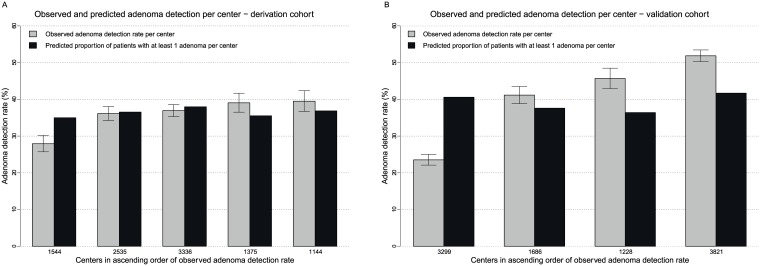
A. Observed adenoma detection rates (including 95% Wilson confidence intervals) versus predicted proportions of patients with ≥1 adenoma per center within A) the derivation cohort (figure at the left) and B) the validation cohort (figure at the right). The centers are ranked in ascending order of adenoma detection rate. The numbers on the x-axis denote the number of patients per center.

Within the derivation cohort the median observed ADR was 35.2% (range: 20.0–52.5%) per physician and 36.9% (range: 27.9–39.5%) per center. Six physicians and no center had an ADR below the recommended threshold[6,7] of ≥25%. The median predicted proportion of patients with ≥1 adenoma was 36.7% (range: 31.0–40.2%) per physician and 36.5% (range: 35.0%-38.0%) per center. The upper bound of the 95%-CI of the observed ADR was lower than the predicted proportion of patients with ≥1 adenoma for five physicians and one center. The lower bound of the 95%-CI of the observed ADR was higher than the predicted proportion of patients with ≥1 adenoma for five physicians and one center (Figs 3 and 4A).

**Fig 5:**
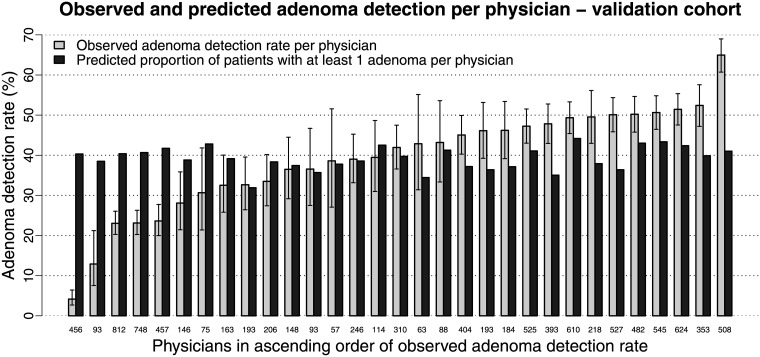
Observed adenoma detection rates (including 95% Wilson confidence intervals) versus predicted proportions of patients with ≥1 adenoma per physician within the validation cohort. The physicians are ranked in ascending order of adenoma detection rate. The numbers on the x-axis denote the number of patients per physician.

Within the validation cohort the median observed ADR was 41.9% (range: 4.2–65.0%) per physician and 43.4% (range: 23.5–51.9%) per center. Five physicians and one center had an observed ADR <25%. The median predicted proportion of patients with ≥1 adenoma was 39.1% (range: 31.9–44.2%) per physician and 39.1% (range: 36.4–41.7%) per center. The upper bound of the 95%-CI of the observed ADR was lower than the predicted proportion of patients with ≥1 adenoma for seven physicians and one center. The lower bound of the 95%-CI of the observed ADR was higher than the predicted proportion of patients with ≥1 adenoma for 13 physicians and three centers (Figs 5 and 4B).

Overall, there was no obvious trend of smaller sample sizes per physician or center at the extremes of observed ADRs. The predicted proportion of patients with ≥1 adenoma varied less than the observed ADR.

### Model performance including physician as predictive factor

Given the moderate discriminative ability of the prediction model, a sensitivity analysis was performed in which the performing physician was added as a variable during model development within the derivation cohort. After stepwise backwards selection performing physician was selected in the model and ethnicity was left out which led to a modest increase in the apparent C-statistic from 0.630 to 0.647.

## Discussion

This study shows that age, sex, BMI, ASA class, surveillance vs. screening, and a Hispanic or Latino ethnicity are independent patient-related predictors of colorectal adenoma detection in a screening and surveillance population. These patient risk factors only modestly account for the variation in individual physicians’ and centers’ ADR. The final multivariable model had a moderate discriminative ability within the derivation and validation cohort, which slightly increased after the addition of the performing physician as predictor. The lowest observed ADRs were lower than predicted, while the highest observed ADRs were higher than predicted. Approximately one in six physicians performed colonoscopy with an ADR below that predicted by patient risk factors.

Our finding that increasing age is an independent predictor of adenoma detection is in line with the recommendation to start screening colonoscopy from 50 years onwards, since adenoma detection increases with age.[35] The increased risk (OR: 1.73, 95%-CI: 1.60–1.88) for adenoma detection in male versus female patients is consistent with the current recommended difference in adenoma detection rate for male patients (≥30%) and female patients (≥20%) that equals an OR of 1.71.[7] Increasing BMI was included as a predictor in almost every previous prediction model for adenoma detection[18,20,24,25] as is the case in our prediction model.

A recent Asian study found a self-reported family history of colorectal adenomas to be associated with an increased risk of adenoma detection.[36] Moreover, a family history of advanced adenomas has been shown to increase the risk of advanced adenoma detection.[37] However in the present study a family history of adenomas was not independently predictive for adenoma detection. Interestingly a family history of CRC was not found to be an independent predictor either, while a family history of CRC has been previously included in prediction models for both adenoma[18,23–25] and advanced adenoma detection.[16–19,22,23]

Some possible predictors for adenoma detection that have been associated with (advanced) adenoma detection or CRC development are not collected within the GIQuIC database. Smoking was included in prediction models for adenoma detection.[18,23–25] Furthermore alcohol consumption has been associated with the development of (advanced) adenomas,[38] and has been included as independent predictor in two prediction models for advanced adenomas.[21,22] Certain medication, such as aspirin has been associated with a decreased risk of CRC development[39] and could therefore have possibly added to the discrimination between patients with and without adenomas.[25] In our model ASA class, which is routinely assessed to make sedation decisions, might function as a proxy for medical condition in general. Nevertheless, assignment of ASA-class might heavily depend on the assessor, and certain medical conditions, if predictive, would be less prone to observer judgment and might be more specific. Lastly, dietary components, e.g. dietary fibers have been associated with a decreased risk of colorectal adenoma development,[40] and fried food, picked food, white meat or green vegetable consumption were predictive of advanced adenoma detection in a Chinese population.[15] However, dietary factor assessment in clinical practice might suffer from recall bias. These factors should be considered to be included as variables within ADR monitoring databases such as GIQuIC, if addition of any of these predictors would improve prediction of adenoma detection.

The moderate discriminative ability of our patient-factor based prediction model for adenoma detection is in line with a recent study in which adjustment of the ADR for age, sex, race/ethnicity, and family history of CRC did reduce the variability in ADRs but had only a small effect on the differences of ADRs between physicians.[41] In another study excluding different patient subgroups from ADR calculations changed ADRs substantially, but had only a small effect on the ADR ranking among physicians[42] suggesting that factors, such as physician-related, procedural or technological factors, are likely to influence the ADR. A physician-effect might also be part of the explanation of the ADR increase during the first ten years of the German CRC screening program, because physicians who started to perform screening colonoscopy detected more adenomas compared to physicians who stopped performing colonoscopies.[43] Unfortunately, no physician-factors were known in our study, therefore their influence on the variation in ADR could not be assessed. Future identification of modifiable physician-factors next to for example withdrawal time could create opportunities for further quality improvement in colonoscopy.

In this study, to the best of our knowledge, we describe the first prediction model for adenoma detection in a screening and surveillance population and its application to compare the predicted proportion of patients with ≥1 adenoma to the individual physician’s and center’s observed ADRs. The study is strengthened by the prospective data collection. The large sample size decreased the risk of model overfitting, which was further decreased by the internal and geographical validation, however, a full external validation is still preferable before the model would be widely applied.

Our study also has some limitations. Firstly, although handled in the most appropriate way with multiple imputations, the substantial proportion of missing data on BMI might have influenced our results. Moreover, data on race and ethnicity was possibly missing not at random and could therefore not be handled with multiple imputation. Data on smoking status was unknown for all patients. Secondly, the race and ethnicity sub classification was based on the possibilities within the GIQuIC form; therefore we could for example not specify whether Asian patients originally came from Asian countries with a low or high colorectal adenoma prevalence. Thirdly, due to the design of data collection the pathologists could not be blinded for all predictors during histology assessment. Fourthly, the number of patients who underwent surveillance colonoscopy earlier or later than recommended is unknown. This could have influenced the ADR in the surveillance group, however compared to screening colonoscopy more patients with ≥1 adenoma were detected during surveillance colonoscopy in the present study. Lastly, since adenoma detection is not considered to be perfect with reported per adenoma miss rates up to 20% of all adenomas,[44] the reference standard for detection, i.e. colonoscopy, is not perfect. If the performing physician or procedural factors are truly influencing the adenoma detection per patient, the outcome assessment might have been biased. Unfortunately, it is not possible to determine to what extent this has influenced the performance of our model and the selection of predictors.

## Conclusions

We developed and validated a prediction model for colorectal adenoma detection in a screening and surveillance population based on patient-related predictors, i.e. age, sex, BMI, ASA class, surveillance as indication, and Hispanic or Latino ethnicity, with a modest discriminative ability. Additional patient-related predictors that were not included in the database, e.g. smoking, alcohol use, medication and medical history, could possibly have added to the model performance. However, these data suggest that variation in ADR between physicians could likely be due to a combination of patient-related and other factors, e.g. physician, procedural or technical issues. These data also suggest that a low individual physician’s ADR, can only partially be explained by patient mix, and efforts to improve ADR to meet current guidelines should be pursued.

## Supporting information

S1 Database De-identified database of the derivation cohort. (CSV)

S2 Database De-identified database of the validation cohort. (CSV)

S1 Data Dictionary Description of the variables within the databases. (XLSX)

S1 File Gastro-Intestinal Quality Improvement Consortium (GIQuIC) data-entry form. (PDF)

S2 File Transparent Reporting of a multivariable prediction model for Individual Prognosis or Diagnosis (TRIPOD) checklist. (PDF)

S1 Table Reasons of high-risk assessment for adenoma detection and adenoma detection rate per high-risk assessment reason. (DOCX)

## Abbreviations

ADR: adenoma detection rate
ASA: American Society of Anesthesiology physical status class
BMI: body mass index
CI: confidence interval
C-statistic: concordance statistic
CRC: colorectal cancer
EQUIP: Endoscopic Quality Improvement Program
GIQuIC: Gastro-Intestinal Quality Improvement Consortium
NA: not applicable
NCT: clinical trial registration number of clinicaltrials.gov
OR: odds ratio
SD: standard deviation
TRIPOD: Transparent Reporting of a multivariable prediction model for Individual Prognosis or Diagnosis
