# Supplementary material for: Development and validation of a prediction model for adenoma detection during screening and surveillance colonoscopy with comparison to actual adenoma detection rates
Source: PLoS One. 2017 Sep 28;12(9):e0185560. doi: 10.1371/journal.pone.0185560 (PMC5619799; doi:10.1371/journal.pone.0185560)
Supplement: S1 File — (PDF) [file pone.0185560.s004.pdf]

S1 File. Gastro-Intestinal Quality Improvement Consortium (GIQuIC) data-entry form

GI Quality Improvement Consortium	GIQuIC
Colonoscopy Data Collection Form	

Patient Sociodemographic Information

**Patient Identifier:**			
**Patient Type:**	☐ Inpatient<br>☐ Outpatient		
**Patient Zip Code:**	☐☐☐☐☐	**Patient Birth Date:**	m m d d y y y y
**Patient Gender:**	☐ Male<br>☐ Female		
Patient Height: (inches)	☐☐	Patient Weight: (pounds)	☐☐☐
**Patient Race:**	☐ American Indian (Native American) or Alaska Native<br>☐ Asian<br>☐ Black or African American<br>☐ Native Hawaiian or Other Pacific Islander<br>☐ White<br>☐ Patient declined to provide<br>☐ Unknown<br>☐ Other		
Patient Ethnicity	☐ Hispanic or Latino<br>☐ Not Hispanic or Latino<br>☐ Patient declined to provide<br>☐ Unknown		
Patient Insurance Type:	☐ Aetna<br>☐ Blue Cross/Blue Shield<br>☐ Cigna<br>☐ Humana<br>☐ United Healthcare<br>☐ Wellpoint<br>☐ Medicare Advantage<br>☐ Medicare Fee for Service<br>☐ Medicaid<br>☐ Tricare<br>☐ None<br>☐ Other (list specific name of plan if not listed above): ____________________________		

GI Quality Improvement Consortium	GIQuIC
Colonoscopy Data Collection Form	

Endoscopy Suite Information

**Endoscopy Facility ID:**	☐☐☐☐	Endo Suite Type:	☐ Hospital<br>☐ ASC/AEC<br>☐ Physician Office
**Physician ID (NPI):**	☐☐☐☐☐☐☐☐☐☐	Endo Suite Teaching Status:	☐ Teaching Facility<br>☐ Non-Teaching Facility
Fellow Physician ID (NPI):	☐☐☐☐☐☐☐☐☐☐	Did the Fellow Physician perform the procedure?	☐ Yes<br>☐ No
Year of Fellowship	☐ Year 1<br>☐ Year 2<br>☐ Year 3<br>☐ Year 4	Physician Specialty	☐ GI<br>☐ IM<br>☐ FP<br>☐ Surgeon<br>☐ Other

General Quality Indicators

**Procedure Date:**	m m d d y y y y
**Endoscopy Procedure:**	☐ Colonoscopy ☐ ERCP<br>☐ EGD ☐ EUS
**Current History & Physical Documented on Chart?**	☐ Yes ☐ No
**Informed Consent Documented in Medical Record?**	☐ Yes ☐ No
**ASA Category:**	☐ ASA I ☐ ASA II ☐ ASA III ☐ ASA IV ☐ ASA V ☐ ASA-E
Sedation type:	☐ None ☐ Moderate ☐ Deep (propofol) ☐ General
Sedation administered by:	☐ Nurse ☐ Endoscopist ☐ Anesthesia professional

Discharge Instructions

***Note**: If the procedure is for an inpatient, please fill out only the questions on Diet Instructions and Medication Resumption. If the procedure is for an outpatient, please fill out all the instruction questions below.*

**Written *Discharge Instructions* provided to patient before discharge?**	☐ Yes ☐ No
Diet Instructions:	☐ Yes ☐ No

**GI Quality Improvement Consortium**	GIQuIC
Colonoscopy Data Collection Form	

Medication Resumption / Orders Given:	☐ Yes ☐ No ☐ N/A
Return to Activities:	☐ Yes ☐ No
Potential Delayed Complications:	☐ Yes ☐ No
Medical Emergency Contact Number:	☐ Yes ☐ No
**Anticoagulation / Anti-platelet Therapy**	
**Anticoagulation / Anti-platelet Therapy: Patient given instructions relative to resumption of therapy**	☐ Yes ☐ No ☐ N/A

**Colonoscopy Procedure Quality Indicators**	
**Colonoscopy Type**:	☐ Colon Cancer Screening ☐ Surveillance ☐ Diagnostic
	If Screening or Surveillance, Year of ***previous colonoscopy***: y y y y
**Bowel Prep Quality:** *(Bowel Prep is adequate if sufficient to accurately detect polyps ≥ 6 mm in size; Inadequate if it is NOT sufficient to accurately detect polyps ≥ 6 mm)*	☐ Adequate ☐ Inadequate

**Colonoscopy Indication – Select at least one (1) reason for performing the colonoscopy**	
☐	Evaluation of unexplained GI bleeding
☐	Unexplained iron deficiency anemia
☐	Screening for colonic neoplasia
☐	Surveillance due to prior colonic neoplasia
☐	Inflammatory bowel disease of the intestine if more precise diagnosis or determination of the extent / severity of activity of disease will influence immediate / future management
☐	Clinically significant diarrhea of unexplained origin
☐	Evaluation of barium enema or other imaging study of an abnormality that is likely to be clinically significant, such as filling defect or stricture
☐	Intraoperative identification of a lesion not apparent/found at surgery (e.g. polypectomy site or bleeding source)

☐	Treatment of bleeding from such lesions as vascular malformation, ulceration, neoplasia, & polypectomy site
☐	Foreign body removal
☐	Excision of colonic polyp
☐	Decompression of an acute nontoxic megacolon or sigmoid volvulus
☐	Balloon dilation of stenotic lesions
☐	Palliative treatment of stenosing or bleeding neoplasms
☐	Marking a neoplasm for localization
☐	Other , specify: ____

***Cecal Landmarks* – Documentation provided in medical record**	
**Ileocecal Valve Photographed**	☐ Yes ☐ No
**Appendiceal Orifice Photographed**	☐ Yes ☐ No
**Terminal Ileum Photographed**	☐ Yes ☐ No

**Colorectal Neoplasm Risk Assessment**	
**Colorectal Neoplasm Risk Assessment for this procedure**:	☐ Average Risk<br>☐ High Risk<br>☐ N/A

If High Risk, select all that apply:

	Colon or Rectal Adenocarcinoma, specify**(c)**:<br>☐ Personal History ☐ Family History (1st degree relative < 60 years old) ☐ Both
	History of Colon Adenoma, specify **(c)**:<br>☐ Personal History ☐ Family History (1st degree relative < 60 years old with advanced adenoma(s))<br>☐ Both
	High Risk Genetic Family Cancer Syndrome (e.g. Familial Adenomatous Polyposis Syndrome, HNPCC/Lynch Syndrome,) **(c)**<br>☐ Personal History ☐ Family History ☐ Both
☐	Advanced Neoplasm (≥ 10 mm, high grade dysplasia, villous component **(c)**

☐	3 or More Adenomas **(c)**
☐	Non Advanced Neoplasm (< 3 adenomas, < 10 mm, no villous component) **(c)**
	Sessile serrated polyp(s) < 10 mm with no dysplasia **(c)**<br>☐ Personal History ☐ Family History (1st degree relative < 60 years old) ☐ Both
	Sessile serrated polyp ≥ 10 mm OR sessile serrated polyp with dysplasia OR traditional serrated adenoma **(c)**<br>☐ Personal History ☐ Family History (1st degree relative < 60 years old) ☐ Both
	Serrated polyposis syndrome* **(c)**<br>☐ Personal History ☐ Family History (1st degree relative < 60 years old) ☐ Both<br>*Based on the World Health Organization definition of serrated polyposis syndrome, with one of the following criteria: (1) at least 5 serrated polyps proximal to sigmoid, with 2 or more ≥ 10 mm; (2) any serrated polyps proximal to sigmoid with family history of serrated polyposis syndrome; and (3) > 20 serrated polyps of any size throughout the colon.
☐	Inflammatory Bowel Disease (≥ 8 years pancolitis or ≥ 15 years left sided colitis) **(c)**
☐	Inflammatory Bowel Disease with Known Dysplasia

**Polyps**	
Number of Polyps Removed During Colonoscopy Procedure:	
Number Polyps Partially Removed During Colonoscopy Procedure:	
Number Polyps Retrieved During Colonoscopy Procedure:	
Polyp Morphology Described:	☐ Yes ☐ No ☐ N/A
Polyp Size Described:	☐ Yes ☐ No ☐ N/A

GI Quality Improvement Consortium	GIQuIC
Colonoscopy Data Collection Form	

Patient Sociodemographic Information

**Patient Identifier:**			
**Patient Type:**	☐ Inpatient<br>☐ Outpatient		
**Patient Zip Code:**	☐☐☐☐☐	**Patient Birth Date:**	m m d d y y y y
**Patient Gender:**	☐ Male<br>☐ Female		
Patient Height: (inches)	☐☐	Patient Weight: (pounds)	☐☐☐
**Patient Race:**	☐ American Indian (Native American) or Alaska Native<br>☐ Asian<br>☐ Black or African American<br>☐ Native Hawaiian or Other Pacific Islander<br>☐ White<br>☐ Patient declined to provide<br>☐ Unknown<br>☐ Other		
Patient Ethnicity	☐ Hispanic or Latino<br>☐ Not Hispanic or Latino<br>☐ Patient declined to provide<br>☐ Unknown		
Patient Insurance Type:	☐ Aetna<br>☐ Blue Cross/Blue Shield<br>☐ Cigna<br>☐ Humana<br>☐ United Healthcare<br>☐ Wellpoint<br>☐ Medicare Advantage<br>☐ Medicare Fee for Service<br>☐ Medicaid<br>☐ Tricare<br>☐ None<br>☐ Other (list specific name of plan if not listed above): ______________________________		

GI Quality Improvement Consortium	GIQuIC
Colonoscopy Data Collection Form	

**Adverse Events**<br>**Please specify immediate adverse events(s) occurring the same day, before the patient leaves the endoscopy facility**	
☐	No Adverse Events
☐	Bowel Perforation
☐	Bleeding (Unplanned Intervention or Hospital Admission)
☐	Emergency Dept visit related to colonoscopy procedure
☐	Hospital Admission related to colonoscopy procedure
☐	Sedation Related (Unplanned Intervention)
☐	Death
☐	Other, specify**:** ____
