# Supplementary material for: Development and validation of a prediction model for adenoma detection during screening and surveillance colonoscopy with comparison to actual adenoma detection rates
Source: PLoS One. 2017 Sep 28;12(9):e0185560. doi: 10.1371/journal.pone.0185560 (PMC5619799; doi:10.1371/journal.pone.0185560)
Supplement: S1 Table — (DOCX) [file pone.0185560.s006.docx]

**S1 Table. Reasons of high-risk assessment for adenoma detection and adenoma detection rate per high-risk assessment reason.**

| **Reasons for high risk assessment** | **Derivation cohort** | | **Validation cohort** | |
| --- | --- | --- | --- | --- |
|  | **All patients** | **Patients with ≥1 adenoma detected** | **All patients** | **Patients with ≥1 adenoma detected** |
|  | **[N=9934]**  **N (%)** | **[N=3568]**  **N (ADR)** | **[N=10034]**  **N (%)** | **[N=4013]**  **N (ADR)** |
| Colorectal adenocarcinoma |  |  |  |  |
| Family history^a^ | 804 (8.1) | 267 (33.2) | 724 (7.2) | 309 (42.7) |
| Colorectal adenoma |  |  |  |  |
| Personal history^b^  Family history^a^  Both | 2322 (23.4)  367(3.7)  119 (1.2) | 1038 (44.7)  123 (33.5)  64 (53.8) | 2764 (27.5)  133 (1.3)  68 (0.7) | 1277 (46.2)  52 (39.1)  36 (52.9) |
| Sessile serrated polyp(s) <10mm without dysplasia |  |  |  |  |
| Personal history  Family history^a^  Both | 41 (0.4)  1 (0.01)  NA | 20 (48.8)  1 (100)  NA | 44 (0.4)  NA  NA | 25 (56.8)  NA  NA |
| Sessile serrated polyp ≥10mm or with dysplasia or traditional serrated adenoma |  |  |  |  |
| Personal history  Family history^a^  Both | 25 (0.3)  NA  NA | 8 (32.0)  NA  NA | 48 (0.5)  NA  NA | 22 (45.8)  NA  NA |
| Serrated polyposis syndrome^c^ |  |  |  |  |
| Personal history  Family history^a^  Both | 2 (0.02)  NA  NA | 1 (50.0)  NA  NA | 5 (0.05)  NA  NA | 2 (40.0)  NA  NA |

ADR, adenoma detection rate: proportion of patients with ≥1 adenoma detected per subgroup; mm, millimeter; N, number of patients; NA, not applicable.

^a^Family history is defined as a first degree relative diagnosed with the condition at an age < 60 years.

^b^All colonoscopies for patients with a personal history of colorectal adenomas have been regarded as surveillance colonoscopies.

^c^World Health Organization definition: 1) ≥5 serrated polyps proximal to sigmoid, with ≥2 ≥10mm; or 2) any serrated polyps proximal to sigmoid with family history of serrated polyposis syndrome; or 3) >20 serrated polyps of any size throughout the colon.
